# The Future of Agriculture is Digital: Showcasting e-Estonia

**DOI:** 10.3389/fvets.2017.00151

**Published:** 2017-09-21

**Authors:** Ene Kärner

**Affiliations:** ^1^Estonian Chamber of Agriculture and Commerce, Tallinn, Estonia

**Keywords:** digitalization, digital agriculture, geographical information applications, livestock performance recording, interoperability, interconnectivity, data ownership

We live in digital society. This is evidenced by the extensive use of information technology in all spheres of life. Digital Society is not just a technology, but a comprehensive social organization, where the information and its exchange play a major role.

In this article, we present successes of Estonia’s digital agenda and argue for coordinated farmer-centered actions for digitization of agriculture at the EU level.

## Main Principles of e-Estonia

Conscious and systematic development of digital society is a strategic choice of Estonia since 1994. When Estonian political and technical leadership began laying the foundation for e-Estonia, it decided on certain principles:

- Decentralization. There’s no central database, and every stakeholder, be it a government department, a ministry, or a business, gets to choose its own system in its own time.
- Interconnectivity. All the elements in the system have to be able to work together smoothly.
- Open platform. Any institution can use the public key infrastructure.
- Open-ended process. As a continuous project to keep growing and improving organically.

## e-Estonia is Based on X-Road and e-ID

The two key ingredients in the infrastructure are the X-Road and e-Identity or e-ID. The mandatory national e-ID card serves as the digital access card for all of Estonia’s e-services, including digital signature, while maintaining the highest level of security and trust.

The X-Road is a critical tool that connects all the decentralized components of the system together. It’s the environment that allows the nation’s various databases and registers, both in the public and private sector, to link up and operate in harmony, no matter what platform they use.

X-Road is the all-important connection between these databases, the tool that allows them to work together for maximum impact. All of the Estonian e-solutions that use multiple databases use X-Road. All outgoing data from the X-Road are digitally signed and encrypted. All incoming data are authenticated and logged.

Originally, X-Road was a system used for making queries to the different databases. Now, it has developed into a tool that can also write to multiple databases, transmit large data sets, and perform searches across several databases (1).

## e-Estonia in Agriculture

Estonian farmers as all Estonian citizens benefit from the usage of e-services. According to a study on the impact of e-services in Estonia (2), users find that the e-services have helped them to save a lot of time, made communication with the government more accessible and easier and have reduced possible errors. For example, time spent on applying for agricultural subsidies at Estonian Agricultural Registers and Information Board decreased from 300 min (filling in forms on paper) to 45 min by filling in an online application. Also, in Estonia, there have been no significant delays in paying out the subsidies. In general, users have saved the most time with e-services, which means that they no longer have to visit various government agencies nor obtain information from a previously separate information system.

Estonian farmers are keen to use new technologies, both in crop and animal husbandry sectors and the benefit of this usually do not raise any doubts. The most significant samples of digital agriculture in Estonia are following.

### Geographical Information Applications

The Estonian public institutions have made serious efforts to develop the GIS systems. Thanks to the X-Road, all the systems are interconnectible and easily combinable *via application programming interface* (APIs). For example, as the databases of Estonian Land Board, E-Land Register, and Estonian Agricultural Registers and Information Board are interconnected, it is easy to find lots of information about any location in Estonian mainland, such as cadastral register number, intended land use, soil type, protected area restrictions, land owner, land user, etc. Unlike in many other countries, these data are open and accessible to public, as the trust and security is assured by access with e-ID. Additionally, the GPS technology enables to track the location and movement of tractors and other mobile machinery, so it is possible to gain full information about activities that are allowed and carried out in this location, also the level of productivity, etc.

Many of these data are practically used in operations of web and mobile applications that are developed for farm management, like VitalFields, eAgronom, Terake.eu. Using these apps has saved substantial amount of time for farmers from paperwork, as filling the fieldbook and compliance reporting for the payment agency are now automatized. Currently, developers focus to real-time data transfer from agricultural machinery into accounting without interim reporting. This would enable significant savings from data processing and more operative access to necessary information for farm management.

### Livestock Performance Recording

In Estonia, the percentage of performance recording of dairy cattle is one of the highest in the world (95% in 2015), approaching rapidly to 100%. Performance recording enables access to data, which is necessary precondition for dairy farm management.

Estonian Livestock Performance Recording Ltd. (ELPR) has created very innovative applications for dairy farmers, which monitor dairy production, milk quality, and animal fertility indicators. Producers can input their data *via* web application, in particular, Vissuke for dairy, Possu for pigs (3). The databases of Livestock Performance Recording Ltd. are interconnected with Estonian Agricultural Registers and Information Board, which enables to synchronize the registration of changes in herd without doubling data entry and minimizes the possible errors. Most of Estonian producers use the ELPR database also for recording herd movement in the accountancy of their enterprises (4).

### Project “Application of Performance Measurement System for More Informed Decision-Making and Increased Efficiency of Production Process at Dairy Farms”

Estonian University of Life Sciences and Estonian Animal Breeders Association have launched a joint project, whose aim is to create a possibility for managers and specialists of dairy farms for benchmarking efficiency of the production process. A special database is created for the project, where participating enterprises enter information about their expenditure and revenues, feed use, and herd movement. Data from ELPR databases are copied automatically in order to avoid double entries. On the basis of these data, the performance metrics (KPI’s) are calculated, which serve as basis for the benchmarking.

One of the project’s main keywords is promptness—data are collected monthly and feedback of the ongoing month’s results is available within 2 months. The time lag compared to getting results from enterprise’s own accountancy is only few weeks. Another significant novelty is the use of Qlik Sense, one of the leading Business Intelligence software packages for analyzing the results. Business Intelligence is highly topical in software development nowadays as it helps to process big data into applicable metrics and reports. Considering the amount of data and limited resources available for average agricultural enterprise, the use of Business Intelligence software might become highly important in the nearest future.

## Technology Only is Not a Solution, Data are Not an Information

The use of technology combined with digital transformation can help farmers to achieve targets in increasing effectiveness and productivity, and to respond to dynamic markets. Nevertheless, the main question is, how to implement digital technologies, and use information produced in the farm management in a most efficient way. Paradoxically, while there are more and more data available to farmers, there are fewer and fewer resources (including management and workforce) to process these data, often because of tense economic and market situation. Solution could be provided by proper guidance and advisory services, but also by using DSS (*decision support systems*), which would liberate farmers from resource consuming data processing.

## Open Data, Interoperability, and Standardization are Crucial

A farm produces many types of data from diverse sources and format. When data are heterogeneous, it is frequently organized in data silos and ends up being separated from other data. Data silos can be created by private companies, public databases, or between states. For small countries like Estonia, avoiding generating data silos at the level of EU Member States is especially important in order to be competitive. Open data, interoperability, and standardization are crucial to avoid data silos. It is also vital to guarantee free access for farmers to public databases. In Estonia, there are well-developed interoperability and interconnectivity between state level Geographic Information systems, but there is still a long way to go to fully open data (5). Interoperability between private companies mostly does not yet exist. For example, data produced in the tractor’s computer are currently not accessible for third parties for using it in different applications as it is protected by license of tractor manufacturer. In case of change, the technology provider, it is impossible to transfer the previous data into new technology. Farmers should be granted appropriate and easy access and be able to retrieve their own data further down the line. They also should not be restricted should they wish to use their data in other systems. Access and data portability should be addressed at EU level, as the farmers, who are often SME-s, might easily be run over in the negotiations with big technology companies. Common understanding of data portability at EU level would also encourage independent software development besides of big technology companies, which would be more flexible and better meet farmers’ needs.

## Details of Data Ownership Must be Discussed Further

Copa-Cogeca, the umbrella organization of EU farmers and agri-cooperatives, have stated that data produced on the farm or during farming operations should be owned by the farmers themselves (6). Farmers must have full control of the use of their personal and private data, also in case when private data can be identifiable in the further data processing. Ownership of aggregated data poses still many unanswered questions, like where exactly is the borderline between “raw” data from individual farm and the new knowledge processed with a specific methodology or algorithm? What to do in the situation when farmer would like to remove the data of his/her farm from the system? How to share the revenue obtained from the aggregated data between the original source (farmer) and the data processor company? These issues need to be discussed further in details with all the potentially interested parts, and common agreement would be favored, preferably at the EU level within the data sharing code of conduct, or coherent strategy of digitalization.

## Digitalization Simplifies the EU Common Agricultural Policy

Apart from filling in the applications and reporting for agricultural subsidies online, an increased use of digitalization, remote sensing, and ICT would improve efficiency, quality, and timeliness of controls and audits. Nowadays, many indicators are precisely measurable and procedures can be automatized, so there is no need to maintain the outdated CAP rules and controls just “for any case.” The most time and resource consuming rules of the CAP should be found out and simplified *via* digital technologies. It would significantly reduce red tape and bureaucracy not only for farmers but also for administrators, both national and European, and every saved hour is a victory for our economy.

## Farmers are the Heart of Digitalization

Finally, it is crucial that farmers and agricultural sector are fully involved to all the discussions about digitalization, which are currently going on in EU and in the world. Launching the strategy and developing of EU common digital market involves many activities and initiatives, which can be useful for farming sector, like Digital Skills and Jobs Coalition Initiative (7). It is very important that the problems and questions mentioned above will be solved while considering the interests of farmers, not only from the point of view of the ICT sector or technology companies. Digitalization of farming sector would contribute to its competitiveness, help to raise farmers’ income, and attract young people to join the traditional activity, which is vital for the whole society.

## Author Contributions

EK made substantial contribution to conception and acquisition of data, wrote the manuscript, checked the references, and acted as corresponding author.

## Conflict of Interest Statement

The author declares that the research was conducted in the absence of any commercial or financial relationships that could be construed as a potential conflict of interest.
